# Peptidergic Systems as Antitumor Targets: A Right Direction to Fight Cancer?

**DOI:** 10.3390/cancers15204975

**Published:** 2023-10-13

**Authors:** Francisco D. Rodríguez, Rafael Coveñas

**Affiliations:** 1 Department of Biochemistry and Molecular Biology, Faculty of Chemical Sciences, University of Salamanca, 37007 Salamanca, Spain; lario@usal.es; 2 Group GIR-BMD (Bases Moleculares del Desarrollo), University of Salamanca, 37007 Salamanca, Spain; 3 Laboratory of Neuroanatomy of the Peptidergic Systems, Institute of Neurosciences of Castilla y León (INCYL), University of Salamanca, 37007 Salamanca, Spain

Undoubtedly, much progress has been made in treating cancer over the past few years, but unfortunately, 28.4 million cancer patients are expected worldwide in 2040 [1]. This data shows the global health burden that cancer represents today and in the future. Therefore, new efforts and investment in antitumor research lines and therapeutic strategies must be urgently addressed to destroy tumor cells specifically.

Peptides and their receptors play a crucial role in cell communication. Notably, tumor cells overexpress peptide receptors when compared with normal cells [2,3], and the expression of peptide receptors (e.g., neurokinin-1 receptor) is involved in the viability of cancer cells [4]. The findings explain the increasing awareness and interest that the scientific community shows regarding the involvement of peptides in cancer development and progression. Its knowledge has significantly increased in recent years since it is well-known that many peptides promote tumor progression (cancer cell proliferation, invasion, migration, antiapoptotic effect, angiogenesis, and lymphangiogenesis) [5]. The same peptide can promote cancer progression in some tumors, but in others, it can exert an antitumor effect [6], and peptide receptor antagonists favor the apoptosis of cancer cells and inhibit their migration, metastasis, and angiogenesis [2]. Together with chemotherapy, peptide receptor antagonists exhibit a synergic antitumor effect and decrease the severe side effects mediated by the cytostatic drugs, considerably alleviating the toxicity promoted by chemotherapy [2,7]. Remarkably, the expression of the peptidergic systems has been associated with poor prognosis, higher tumor aggressiveness, increased relapse risk, and tumor size [8].

The overexpression of the same receptor in different types of tumors allows for similar antitumor strategies by, for example, administering a peptide receptor antagonist alone or combined with chemotherapy/radiotherapy. The high expression of peptide receptors in tumor cells could also be helpful for cancer diagnosis (tumor biomarker) and treatment after implementing antitumor strategies such as the use of peptide receptor antagonists [2], cytotoxic peptide conjugate-based cancer therapy (e.g., vasoactive intestinal peptide-ellipticine conjugate), or peptide receptor radionuclide therapy (PRRT) [9,10]. For example, the PRRT clinical applications targeting the somatostatin receptor have been reviewed by the group of Christophe M. Deroose from the University Hospitals Leuven (Leuven, Belgium) [11]. This therapy consists of dispensing a tumor-targeting radiopharmaceutical into the bloodstream. Afterward, the binding and holding of the radiopharmaceutical to a specific peptide receptor promotes lethal DNA damage in cancer cells [11]. This review focused on targeting the somatostatin receptor (overexpressed, for example, in neuroendocrine tumors, neuroblastoma, medullary thyroid cancer, and meningioma) by using PRRT. The authors described PRRT basic principles and PRRT potential clinical applications [11]; they also discussed the efficacy of PRRT, the PRRT patient outcome impact, the PRRT toxicity induced by several pharmaceuticals, and PRRT optimization strategies such as combination therapies and modifications of receptor ligands (tandem and duo PRRT, chemoPRRT, targeted molecular therapy, radiosensitizers, or somatostatin analogs), administration route (intravenous, intra-arterial), type of radionuclide, dosimetry, and the introduction of novel radiopharmaceuticals (alpha emitters) and new vector molecules (somatostatin receptor antagonists) [11]. Additionally, the combination of PRRT with radiosensitizers may improve treatment efficacy. The association of inhibitors of PARP (poly-[ADP-ribose]-polymerase), an enzyme essential for reparation of single-strand DNA breaks, with ^177^Lu-DOTA-octreotate significantly increased cell death in preclinical studies [12]. It is important to comment on the numerous PRRT optimization strategies and the standardized PRRT scheme proposal suggested by the authors, as well as the exhaustive review of the PRRT clinical studies currently performed. The authors claimed that the available data supports the claim that PRRT is a validated strategy for treating patients suffering from advanced neuroendocrine tumors and that trials testing the optimization strategies proposed in the review are awaited to ameliorate PRRT treatment efficacy [11]. Finally, they established ten lessons learned from the PRRT development that will help accelerate the future development of radionuclide therapy [11]. PRRT tumor-targeting strategies require profound knowledge of the selected receptor functional states, the definition of ligand orthostatic sites, the potential allosteric regulations, possible biased effects upon ligand binding, receptor internalization rates, and intracellular targets. This knowledge will allow more precise design of conjugation methodologies and carriers to obtain more suitable, specific, and effective ligands.

It has been shown that peptides also display an antitumor effect, for example, by blocking cell proliferation and angiogenesis [5,9]. The use of glucagon-like peptide-1 receptor agonists (GLP-1 RAs) to treat hepatocellular carcinoma (HCC) has been reviewed by the group of Georgios Germanidis from the Aristotle University of Thessaloniki (Thessaloniki, Greece) [13]. GLP-1, released by L-cells of the proximal colon and small intestine, regulates glucose-dependent insulin secretion by beta-pancreatic cells. Given the essential participation of GLP-1 in glucose homeostasis, GLP-1 receptor agonists (GLP-1 RAs) play a crucial role in treating type 2 diabetes and obesity. In addition, the data reported in the review also demonstrated that GLP-1 RAs control the molecular pathways involved in HCC genesis and progression, such as tumor cell proliferation, oxidative stress, and inflammatory processes [13]. The authors reviewed several studies in which GLP-1 RAs (exendin-4 and liraglutide) exerted an antitumor action against HCC. These peptide agonists blocked hepatocarcinogenesis, inhibited tumor cell growth, promoted apoptotic mechanisms in cancer cells, favored autophagy in HCC cells, and increased natural killer cell-mediated oncolytic activity [13]. For this reason, the authors formulated the following interesting question: How far beyond diabetes can the benefits of GLP-1 RAs go [13]? They also advised that future research lines must be explored; for example, how GLP-1 RAs could act in combination therapy with chemotherapy and which gastrointestinal side effects could appear in high-risk individuals after GLP-1 RA administration [13]. The review is a clear example of the therapeutic potential of peptides as antitumor agents and how they can exert unexpected antitumor effects. In the same line of reasoning, peptide receptor antagonists currently and exclusively used as antiemetics in clinical practice also display unexpected antitumor actions. This is the case of the drug aprepitant (a neurokinin-1 receptor antagonist), which exerted an antitumor effect against many kinds of cancer. For this reason, the repurposing of this antiemetic drug as an antitumor agent has been suggested [2]. Aprepitant is also a cough suppressant. However, it has been found that the drug displayed an antitumor action at higher doses than those suppressing cough. Hence, receptor occupancy is a fundamental factor demanding due consideration [14].

Other encouraging strategies, including the use of cell-penetrating peptides (CCPs) [15] and inhibitors of hypoxia-inducible transcription factors (HIFs) [16], represent valuable tools as therapeutic and diagnostic agents. The implication of CCPs in cancer has been reviewed by Ryan A. Bottens and Tohru Yamada from the University of Illinois (Chicago, IL, USA) [15]. CCPs are short peptides that translocate through the cell membrane carrying large cargo biomolecules (i.e., proteins, RNA, DNA, or bioluminescent molecules) with therapeutic and diagnostic potential [15]. Specific delivery may be achieved by taking advantage of distinctive molecular changes occurring in tumor cells compared with healthy cells. A characteristic of some tumor cells is that plasma membrane anionic phosphatidylserine can flip-flop, facilitating a negatively charged external surface that enables the internalization of cationic peptides. The authors reviewed the types of CPPs (i.e., cationic, anionic, and amphipathic). In addition, they discussed the capacity of CCPs to target specific organelles, increasing the specificity of the delivery so that toxicity can be reduced and therapeutic efficacy can be improved [15]. They showed the current CPP-guided organelle-targeting procedures for the endoplasmic reticulum, mitochondria, lysosome, endosome, and nucleus. They had an in-depth discussion regarding the use of CCPs for cancer diagnosis and antitumor treatment. In addition to the cell-penetrating ability, the authors stated that some CCPs show intrinsic anticancer effects. Hence, combining CCPs with anticancer properties with specific antitumor cargos potentiates the fight against tumor cells at several levels [15]. Finally, the authors reviewed and discussed the clinical trials (e.g., RI-TAT-p53C′, DTS-108, p28) in which CCPs have been tested and highlighted the limitations in the use of CCPs, such as in vivo stability, protease inactivation, penetrating efficacy, immunogenicity, and cell selectivity [15]. Advances in peptide chemistry to generate new CCPs exhibiting more metabolic stability, productive actions, and less toxicity could overcome these limitations.

Ilias Mylonis, Georgia Chachami, and George Simos from the University of Thessaly (Larissa, Greece) reviewed the participation of HIFs (hypoxia-inducible transcription factors) in cancer and the effects mediated by HIF inhibitors, including inhibitor peptides [16]. HIFs are heterodimeric proteins formed by an oxygen-inducible subunit (i.e., HIF-1α, HIF-2α, and HIF-3α) and a constitutively expressed HIF-1 monomer (or ARNT) [17]. The HIF family of proteins is activated and translocated to the cell nucleus in the absence of oxygen. However, when oxygen is abundant, HIF prolyl hydroxylation facilitates inactivation by ubiquitination and hydrolysis within the proteasome complex. Oxygen is not the only regulator of HIFs. Transcription, translation, and posttranslational modifications also represent important mechanisms governing HIF activity. HIFs control many genes involved in angiogenesis, erythropoiesis, and metabolism; their overexpression has been associated with cancer development. Moreover, HIFs promote the epithelial-to-mesenchymal transition and the resistance to radiotherapy/chemotherapy, and HIF-1 regulates cancer cell invasion and metastasis, genomic instability, and the anticancer immune response [16]. Accordingly, HIF inhibition represents a promising antitumor strategy. The inhibitory approach can be performed by applying peptides mimicking HIF domains involved in protein-protein interactions, which are essential for their activity. These HIF inhibitors impede the association of HIFs with cell components necessary for the activation of HIF [16]. The authors discuss the peptide inhibition of HIF heterodimerization, the peptide inhibition of HIF-dependent transactivation, and the inhibition of HIF-1α nuclear accumulation and nuclear interactions [16]. Non-peptide and peptide molecules have been analyzed to annihilate HIF interactions with other proteins and DNA domains. Blocking of HIF activity by peptides relies on the design of modeled aminoacidic sequences similar to specific HIF contacting sites that may behave as a decoy for the functional candidate protein or DNA sequence. This approach, however, requires a profound and detailed knowledge of the HIF interactome and the specificity and kinetics of such transactivation and regulatory contacts [16]. For example, one interaction that allows the association of HIF-α isoforms with ARNT to achieve functional heterodimers may be hampered by sequestering ARNT or by blocking the interaction with the PAS heterodimerization domain [16]. The administration of peptides has serious drawbacks, limiting their therapeutic potential because their plasma half-life is short and their cell permeability and oral bioavailability are poor. For these reasons, the authors suggested obtaining HIF peptide inhibitors that share some of the advantages of HIFs as small chemical inhibitors, such as permeability, pharmacokinetics, oral delivery, and half-life [16]. Peptide cyclization, elimination of catalytic sites, co-administration of peptides with protease inhibitors, covalent modifications in the amino acid sequence, peptide-loaded nanoparticles, absorption enhancers, and the conjugation of peptide drugs to synthetic or natural polymers represent current strategies to enhance their therapeutic potential (i.e., cell-targeting peptides and cell-permeable peptides), stability, and delivery [9,10].

Because the peptidergic systems are involved in cancer development, molecular targets (e.g., peptide receptors) and ligands (e.g., peptide receptor antagonists, agonists, and biased agonists) that destroy tumor cells are promising antitumor research lines that must be potentiated. Cancer cells’ overexpression of peptide receptors opens the door to developing antitumor therapeutic strategies. Overexpression may also serve as a tumor biomarker/diagnostic tool. The findings reported above confirm the crucial role of peptides and their receptors in cancer and raise novel antitumor therapeutic strategies that could be applied in clinical practice and require urgent development [11,13,15,16]. In sum, an increasing number of findings confirm that the peptidergic systems are therapeutic targets for cancer treatment, and hence, novel possibilities for translational research arise. This editorial aims to stimulate the study, increase the knowledge of the peptidergic systems’ vital role in cancer, and promote the scientific interest of research in academia and the pharmaceutical industry.
